# A new approach to designing easily recyclable printed circuit boards

**DOI:** 10.1038/s41598-022-26677-y

**Published:** 2022-12-23

**Authors:** Dmitriy Khrustalev, Arman Tirzhanov, Anastassiya Khrustaleva, Marlen Mustafin, Azamat Yedrissov

**Affiliations:** 1 Medical University of Karaganda, 100000 Karaganda, Kazakhstan; 2 Karaganda Economic University of Kazpotrebsouz, 100000 Karaganda, Kazakhstan; 3 Nazarbayev University, 010000 Nur-Sultan, Kazakhstan

**Keywords:** Environmental chemistry, Electrical and electronic engineering

## Abstract

Due to the ever-increasing amount of electronic waste (e-waste) worldwide, the problem of the effective disposal of printed circuit board waste (WPCB), which are environmentally hazardous, difficult to recycle and economically valuable products, has become a major environmental challenge. Conventional WPCB recycling techniques have low efficiency and require tough processing, such as heat treatment and high pressure. This paper presents a new composite material for the manufacture of printed circuit boards (PCB) that can be easily recycled into their original components and reused. In addition, the most valuable PCB components (electronic components containing precious metals) can be easily separated from the printed circuit board and reused. This study demonstrates the benefit of using biodegradable polymers as binders for PCBs in terms of environmentally friendly and efficient recycling.

## Introduction

The rapid growth in the use of electronics in various devices, both for household use and electronic devices for monitoring various processes, has led to a steady increase in PCB production. Ultimately, this has led to an increasing amount of obsolete and unusable circuit boards^1^. According to statistics, more than 50 million tons of e-waste are accumulated in the world every year, and up to 10% of this mass is WPCB^2^.

PCB traditionally used in the electronics industry consists of a composite, dielectric base that acts as a rigid, mechanical frame. Electrically conductive tracks are made by etching copper foil formed on one or both sides of the dielectric base. The dielectric base consists of several layers of glass fabric or paper impregnated with thermosetting resin as a binder and then formed in a hot press^3^. Currently, highly toxic raw materials (epoxy and phenol–formaldehyde resin and their mixtures; combined epoxy-silicone resin; combined epoxy-polyimide resin, bismaleimide resins, triazine resin, etc.) are used as binders. These resins are derived from non-renewable sources. In addition, PCBs made from these resins are not degraded by microorganisms under environmental conditions, which contradicts modern requirements for the safety of chemical processes and materials^4^.

WPCB consisting of metallic (~ 30 wt%) and a non-metallic fraction (~ 70 wt%)^5^ are the most difficult to recycle, hazardous and valuable components of electronic waste^6^. Despite the diverse applications of printed circuit boards, from mobile phones and household appliances to automobiles and industrial processes control systems, WPCBs are characterized by a relatively high content of precious metals Pd, Au, Pt, Ag and base metals such as Cu, Fe, Ni, Zn, Sn, Pb. Moreover, even in the same type of products (e.g. mobile phones), the content of metals can vary by more than tenfold^7^. From an economic point of view, the processing of precious metals is very promising as each ton of WPCBs contains on average 130 kg of copper, 1.38 kg of silver, 0.35 kg of gold and 0.21 kg of palladium, wherein precious metals can account for more than 80% of the economic value^8^.

Today, WPCB recycling is mainly aimed at recovering high value-added metals, while the non-metallic fraction is usually landfilled or incinerated without further recycling. The non-metallic WPCB fraction contains toxic resins and brominated flame retardants^9^, which are extremely hazardous compounds affecting human health and causing cancer^10,11^. It is worth noting that toxic WPCB compounds can easily enter groundwater from landfills, leading to long-term contamination of vast areas^12^. The above threats have prompted an active scientific search for WPCB disposal^13–16^ and recycling methods^17–20^.

Currently, the rapid development in the synthesis and production of new biodegradable polymers is stimulating scientists to develop new types of binders derived from renewable raw materials produced through biotechnological and chemical processes^21^. In particular, the study of biodegradable polymers draws specific interest due to their increasingly diverse applications. Biodegradable polymers are widely applied in packaging and medicine, and the fields of their practical application are expanding significantly^22–24^.

Polymers based on polylactic acid (PLA) and copolymers of PLA with other hydroxycarboxylic acids are thermoplastic polymers with mechanical and electrical properties close to those of thermosetting resins, but at the same time, these materials can be easily recycled through chemical and biotechnological processes for reuse^25^.

In this work, we present a new approach for using biodegradable polymers as binders for PCB fabrication. The use of biodegradable and easily recyclable polymers as binders for PCB manufacturing opens new perspectives for both environmental protection and resource conservation and aims to maximize the recovery of valuable materials for their reuse.

## Experimental

### Materials

A high molecular weight polycrystalline PLA (140 kg mol^–1^) with an optical purity above 99% was purchased from Luhua (China). The glass transition and melting points of 65 °C and 180 °C, respectively. PLA was used without any additional purification. Tetrahydrofuran (99.8%), acetone (99.5%), ethyl acetate (99.8%), and iron (III) chloride (97%) were purchased from Sigma Aldrich (USA). For PCB fabrication, plain weave glass fiber designed for PCB fabrication and 18 μm copper foils purchased from CN-FT JOVI Technology and Trading Co., Ltd. (China) were used.

### Characterizations

#### Scanning electron microscopy (SEM)

The structure of the composite material PCB was studied with a MIRA3 TESCAN scanning electron microscope (Brno, Czech Republic) at an accelerating voltage of 3.0 kV.

#### Fourier transform infrared spectroscopy (FTIR)

FTIR spectra were recorded in the frequency range 500 cm^–1^ to 4000 cm^−1^ on a Thermo Nicolet iS10 FTIR spectrometer (Waltham, USA).

#### Differential scanning calorimetry (DSC)

DSC investigation of the glass transition temperature of composite materials was carried out with a Simultaneous Thermal Analyzer (STA) 6000, Perkin Elmer (Waltham, MA, USA). The samples were heated from 25 to 400 °C at a rate of 10 °C/min. The tests were carried out in a nitrogen atmosphere with a nitrogen flow rate of 60 ml min^–1^.

#### Tensile strength tests

Tensile strength tests were performed on the electromechanical materials testing machine Tinius Olsen H25KT (Redhill, Surrey, England), according to the ASTM D638 standard. The samples were plates of ~ 1.0 mm thickness, 100 mm length, 10 mm width and ~ 3.0 mm working area width. The test was carried out at a temperature of 23 °C and relative humidity of 50%. The loading rate of the samples was 5 mm/min ± 1%. At least seven specimens of each material were tested and the average values were calculated. Tensile strength was determined using the equation:

$${\sigma }_{p}=\frac{{P}_{max}}{{S}_{0}}$$

where P_max_-maximum load preceding the specimen failure, N. S_0_ = b^.^h-an initial cross-section of the sample, mm^2^; b, h-the width of the working area and the thickness of the sample respectively, mm.

#### Bending strength tests

The bending strength tests were performed according to the three-point method on a Computer Control Electronic Universal Testing Machine, WDW-3, HST (Jinan, China), in accordance with ASTM D7264 standard. The specimens were rectangular plates of h = 1.0 mm thickness, b = 10 mm width and 100 mm length. The test was conducted at 23 °C and 50% relative humidity. The test machine provided a uniform speed of relative motion of the loading tip and the support. An error of the measurement was ± 0.5%. The bearing and tip converged at a constant speed of 5 mm/min. Specimens were loaded with a single tip with a force P applied at the center of the specimen between the supports. At least seven specimens of each material were tested and the average values were calculated. The following equation was used to calculate the bending strength:

$$\upsigma =\frac{3{P}_{max }\mathrm{L}}{2\mathrm{b}{h}^{2}}\left\{1+6{\left(\frac{\mathrm{v}}{L}\right)}^{2}-4\left(\frac{\mathrm{v}h}{{L}^{2}}\right)\right\}$$

where σ—stress at the outer surface in the load span region, MPa; P_max_—maximum load preceding the specimen failure, N; L—support span, mm; b—width of the beam, mm; h—thickness of the beam, mm; v—deflection value of the specimen, in the middle between the supports, mm.

#### Dielectric characteristics

The dielectric characteristics of PCBs were studied using an Aktakom AM-3001 Digital LCR meter, T&M Atlantic (Miami, Florida USA), at 23 °C, under normal conditions. Each material was tested on five samples and the average values were calculated.

A Schulze Blue Press X Pneu (Schulze GmbH, Germany) thermal transfer press was used to make the PCB laboratory samples.

## Results and discussion

### Fabrication of a double-sided metallized printed circuit board and installation of electronic components

**Figure 1:**
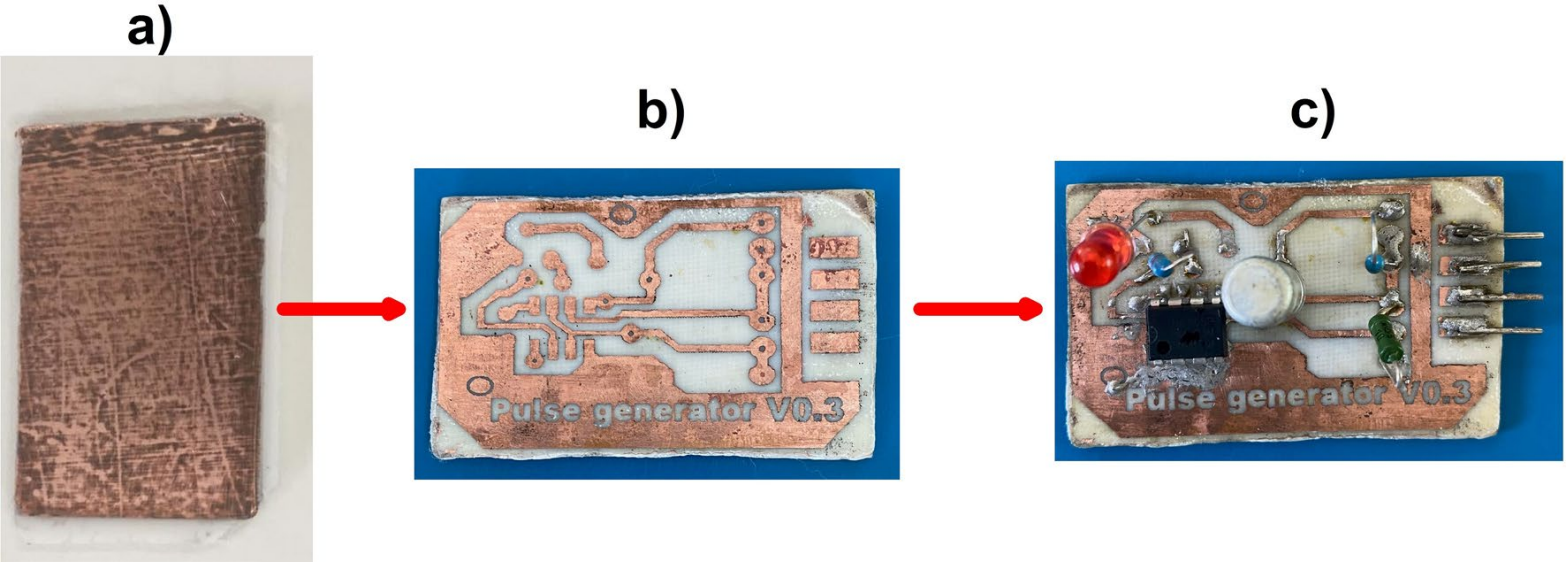
PCB fabrication and installation of electronic components: (**a**) Laboratory fabricated double-sided metalized PCB; (**b**) PCB with conductive copper tracks; (**c**) Electronic device fabricated in the laboratory.

To prepare the prepreg (composite PCB backbone), PLA was dissolved in chloroform at 75–80 °C and a molar ratio of PLA to chloroform of 1:3. The solution was prepared in a reflux condenser with constant heating and stirring on a magnetic stirrer at 200 rpm. Then, sheets of glass fabric 60 mm × 110 mm in size were dipped into the obtained solution and dried at room temperature in a chemical cabinet for 2 h. The density of the prepreg obtained was 110–140 g/m^2^. To make a printed circuit board, 6 sheets of prepreg were stacked on a metal mold covered with Teflon. The copper foil was placed on the bottom and the top of the prepreg stack. After that the mold was closed with a metal plate (100 mm × 120 mm) and also covered with Teflon, to avoid the adhesion of the composite material to the molds. The mold was then placed into a Heat Press and heated to 195 °C. After the prepreg had softened for 5 min, a pressure of 0.2 MPa was applied to the mold for 1 min. The mold was removed from the press and after cooling to room temperature, the double-sided metalized PCB with a thickness of ~ 1.0 mm was removed from the mold (Fig. 1a).

Conductive tracks were applied to the resulting PCB with a special marker (Edding 780). The excess copper foil was etched in iron (III) chloride solution (500 g/l) at 50 °C for 20 min. After the etching process, the marker traces were thoroughly cleaned with ethanol and a PCB with conductive copper tracks was obtained (Fig. 1b). Next, the electronic components (chips, LEDs, capacitors, and resistors) were soldered to the PCB with the Rose alloy (melting point 95 °C) (Fig. 1c). The reason for choosing Rose alloy was the fact that at soldering temperatures above 120 °C, the copper tracks sometimes exfoliated from the composite base.

### Measurements of dielectric, thermal and mechanical properties of PLA-PCB

**Figure 2:**
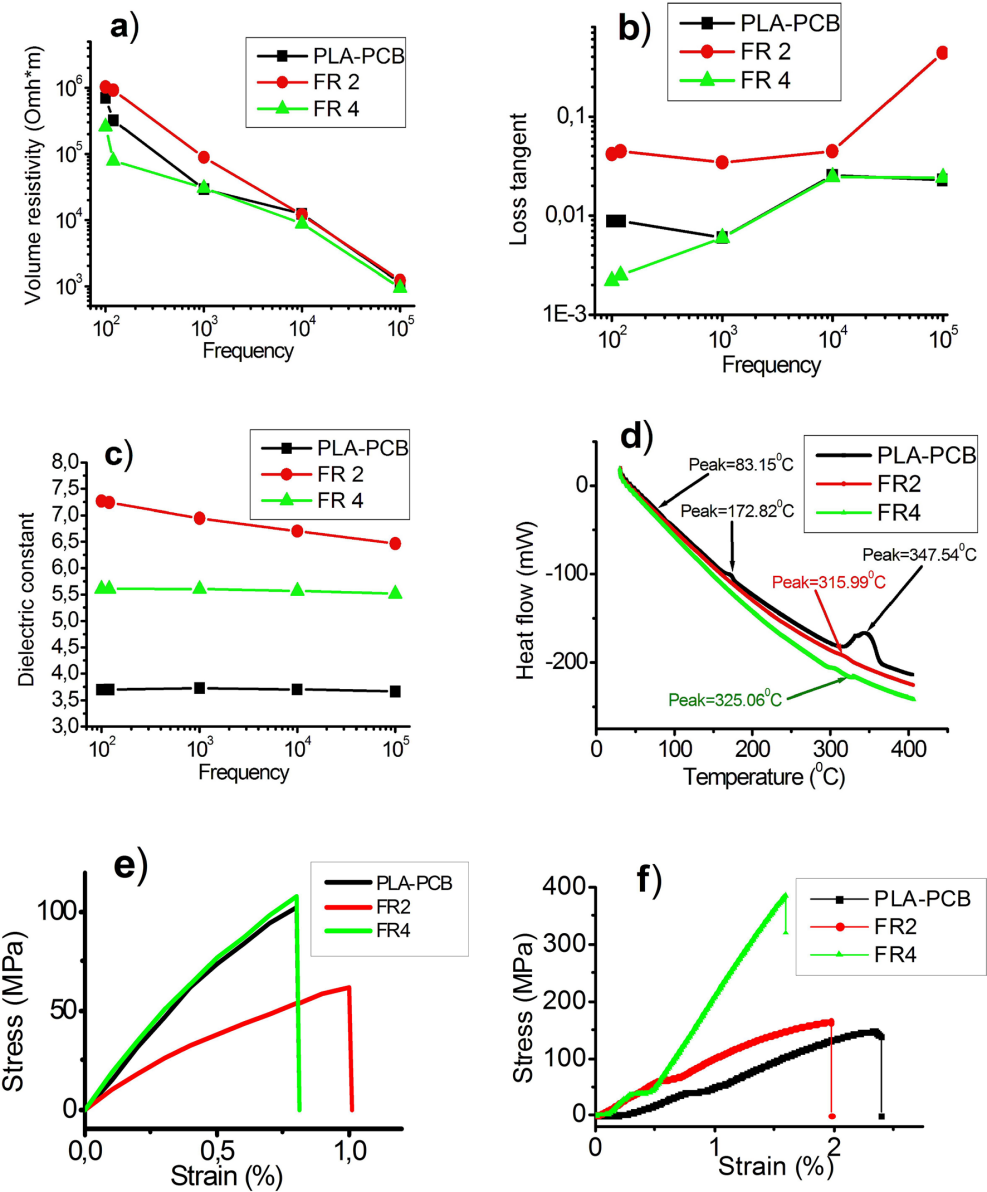
Permittivity measurement results of three PCB plates: (**a**) volume electrical resistivity, (**b**) the loss tangent values, (**c**) dielectric permittivity, (**d**) thermogravimetric-differential scanning calorimetry, (**e**) ultimate tensile strength, and (**f**) flexural test.

Printed circuit boards that are used in the electronics industry are subject to a variety of requirements depending on the area of application. However, the list of the most important properties of a composite dielectric PCB base includes such parameters as volume resistivity, the loss tangent values, dielectric permittivity, glass transition temperature, tensile strength and bending strength. To compare the electrical properties of the most common commercial PCBs (FR2, FR4) and lab-made PLA-PCB, we performed comparative measurements of these properties, which are shown in Fig. 2.

A brief comparison of the main dielectric, thermal and mechanical characteristics of PLA-PCB and commercial PCB samples are presented in Table 1.

**Table 1:** Dielectric, mechanical and thermal characteristics of PLA-PCB, FR2 and FR4.

Material Property	Unit	PLA-PCB	FR2	FR4
Volume resistivity at 10^5^ Hz	Ohm*m	1.12·10^3^	1.22·10^3^	0.79·10^3^
Loss tangent values at 10^5^ Hz	–	2.89·10^–2^	4.37·10^–1^	2.42·10^–2^
Dielectric permittivity at 10^5^ Hz	–	3.66	6.46	5.51
Glass transition temperature T_g_	°C	83.1	65	134.5
Ultimate tensile strength	MPa	102.3	61.9	108.1
Ultimate bending strength	MPa	147.6	162.4	321.5

The results show that the experimental values of volume resistivity, loss tangent values, dielectric permittivity, glass transition temperature, tensile strength, and bending strength of PLA-PCB are generally equal to those of commercial samples FR2 and FR4. The volume resistivity at 10^5^ Hz of PLA-PCB is higher than the FR2 values and lower than the FR4 values. Loss tangent values at 10^5^ Hz PLA-PCB are lower than FR2 and almost equal to FR4. The dielectric permittivity of PLA-PCB at 10^5^ Hz is lower than that of commercial PCBs. The glass transition temperature of PLA-PCB is between FR2 and FR4. The ultimate tensile strength of PLA-PCB is higher than FR2 and practically equal to the ultimate tensile strength of FR4. The ultimate bending strength of PLA-PCB is lower than that of FR4 and slightly lower than that of FR2, which, in our opinion, is due to the fact that FR4 the number of fiberglass layers is more than 8, whereas PLA-PCB contains just 6 layers of fiberglass.

### Recycling a lab-made printed circuit board

**Figure 3:**
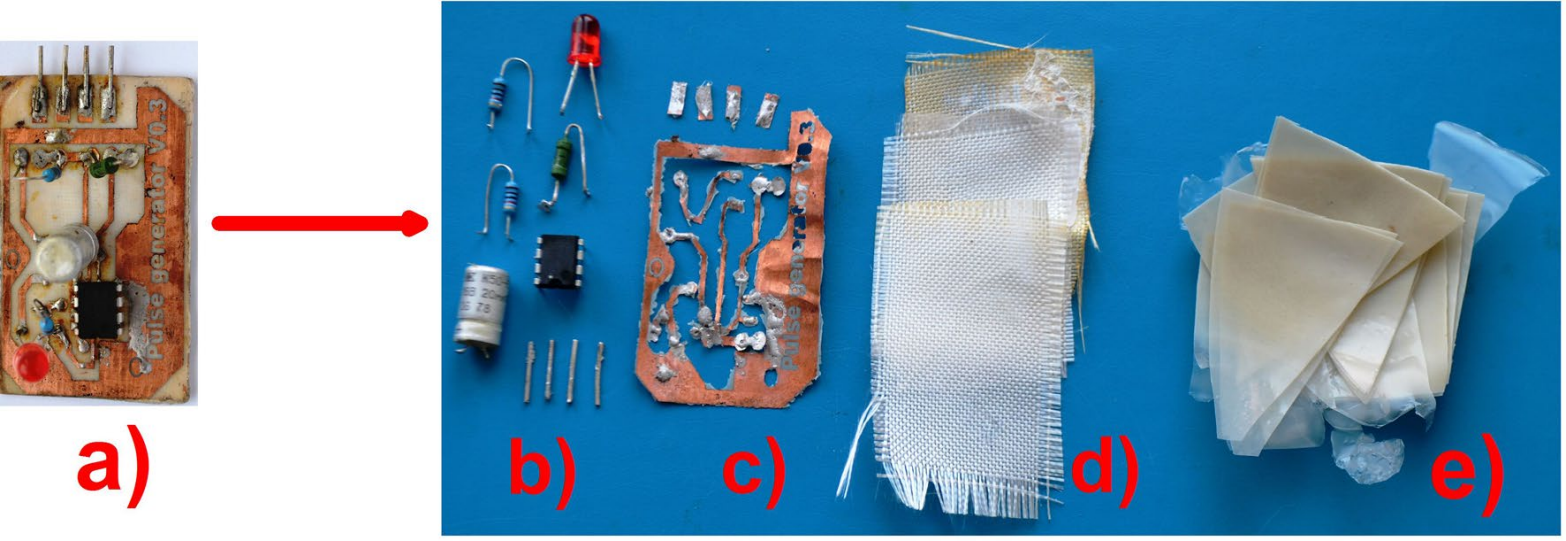
PCB recycling: (**a**) lab-made PLA-PCB; (**b**) electronic components; (**c**) copper tracks; (**d**) fiberglass; (**e**) polylactic acid, after the recycling process.

The choice of solvent was determined based on the recommendations of the Innovative Medicines Initiative (IMI)-CHEM21^26,27^ which summarized the safety analysis of solvents used in the pharmaceutical industry. The safety of solvents was evaluated according to the following criteria: acute toxicity and chronic toxicity for humans; environmental hazard; boiling point and flash point. Based on this evaluation, the possibility of using acetone, ethyl acetate, tetrahydrofuran and chloroform for PLA-PCB disposal was tested in this work. In this series of solvents, chloroform is the most effective. However, due to its high carcinogenic properties, chloroform has been excluded from the list of solvents for PLA-PCB disposal. The worst solvent was ethyl acetate, which did not dissolve the test samples under ultrasonic conditions. PLA samples were dissolved in acetone within 30 min and in tetrahydrofuran in less than 8 min. Thus, tetrahydrofuran appeared to be the most efficient solvent for PLA-PCB disposal. Tetrahydrofuran is not classified as a “hazardous” solvent but rather a “problematic” one. At the same time, tetrahydrofuran has no carcinogenic effect and is not prohibited for use in the pharmaceutical industry for the manufacture of medical devices^26,27^. In addition, considering that tetrahydrofuran can be easily distilled from PLA and reused, this solvent was used in our experiments. The recycling process for lab-made PCB is shown in Fig. 3. For sample recycling, the PLA-PCB (Fig. 3a) was placed in a container of solvent tetrahydrofuran and placed in an ultrasonic bath. This method allowed complete separation of the binder (PLA), copper tracks with electronic components and filler (fiberglass) from each other, without using additional manual^28^, mechanical and thermal processes^29^. The process of the electronic device recycling was completed completely in 30 min. The PLA solution in tetrahydrofuran was evaporated to dryness, in a rotary evaporator in a vacuum at a water bath temperature of 40 °C, resulting in 98 wt% of the PLA being recovered (Fig. 3e).

After recycling, only copper tracks (Fig. 3c) with electronic components (Fig. 3b) and fiberglass (Fig. 3d) remained in the container. Figure 3 shows the electronic components after they have been separated from the copper tracks.

Previously, an article^19^ showed the possibility of PLA-PCB recycling under extraction conditions in a Soxhlet apparatus, in which ethyl acetate dissolved PLA and separated it from the filler and the copper tracks. Despite the promising results, the fundamental disadvantage of recycling in Soxhlet extraction is its duration and energy consumption. In contrast, PLA-PCB recycling with ultrasonic sanitation is twice as fast as in the Soxhlet apparatus^19^ and energy-efficient. It should also be noted that, unlike Soxhlet extraction, ultrasonic sanitation does not require running water for cooling.

It is noteworthy that the marking and protective coatings on the microchip, resistors, or capacitors did not disintegrate and their electrical properties were fully intact (Fig. 3b), allowing expensive electronic components for their entire life cycle.

Tetrahydrofuran was recovered after the PLA-PCB recycling process by solvent distillation on a rotary evaporator under a vacuum in a water bath. After solvent distillation and vacuum drying, PLA was extracted (Fig. 3e).

**Figure 4:**
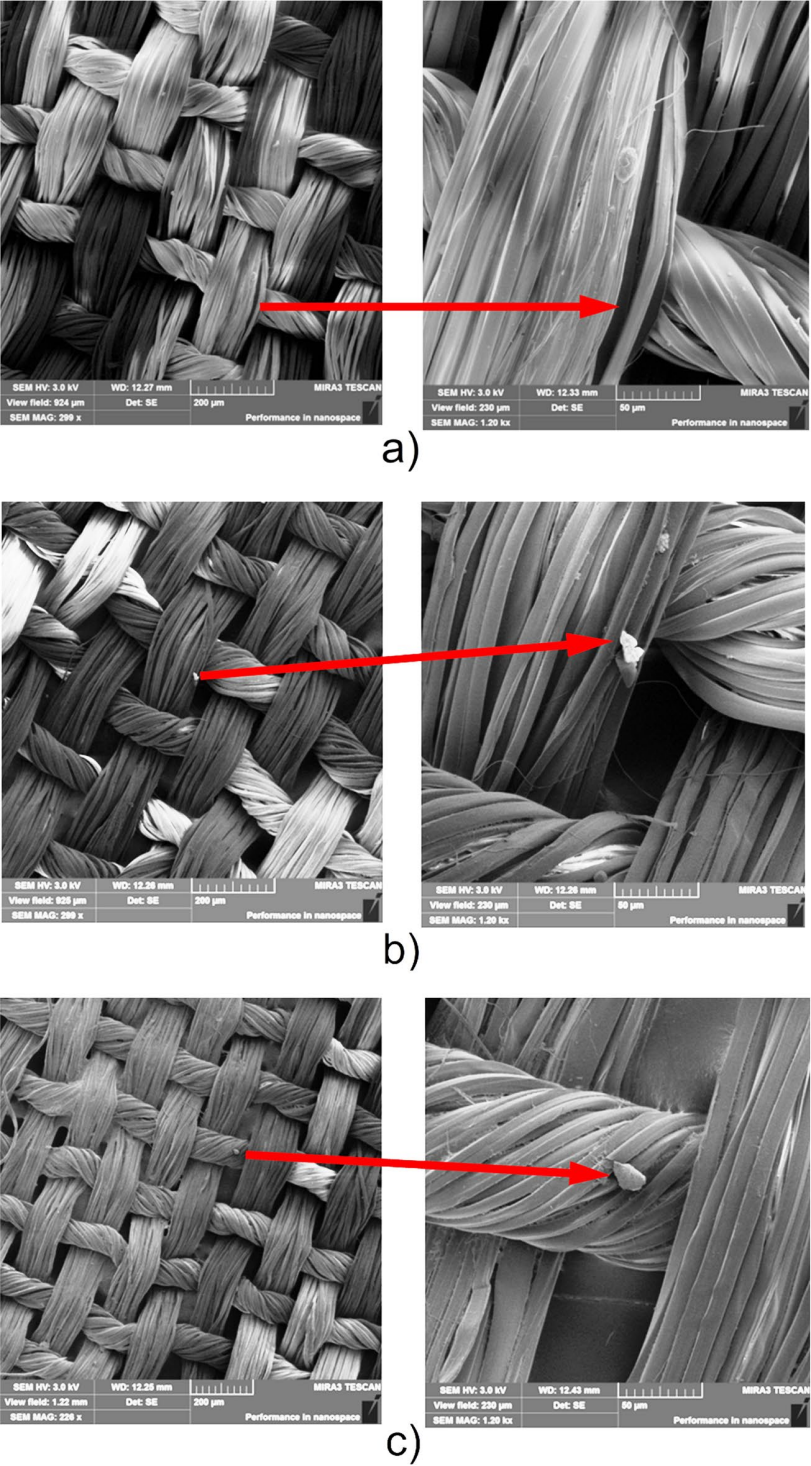
SEM images of glass fabrics: (**a**) initial glass fabric used for PLA-PCB fabrication; (**b**) glass fabric after single recycling; (**c**) glass fabric after triple recycling.

In order to examine the complete dissolution of the PLA, the fiberglass surface was examined with a scanning electron microscope. SEM images of the original fiberglass and the fiberglass after recycling are shown in Fig. 4. After recycling, the binder (PLA) dissolved completely and the fiberglass fabric was intact (Fig. 4b). Even after recycling three times, the fiberglass remained intact (Fig. 4c), indicating a high probability of reuse, whereas, in traditional WPCB recycling techniques, the fiberglass is damaged by thermal, mechanical and chemical processes and can only be reused in building blocks as a reinforcing filler^30^.

**Figure 5:**
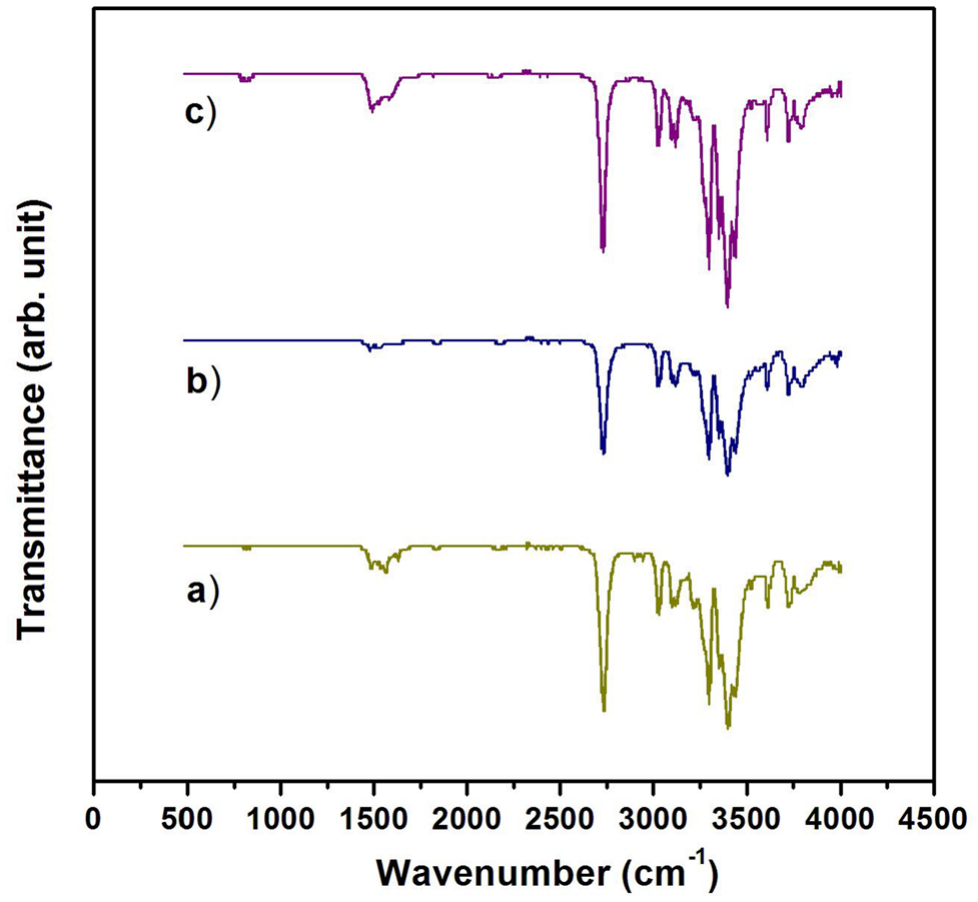
FTIR spectra of the PLA: (**a**) Initial; (**b**) once and (**c**) three times reconstituted from recycled PLA-PCB boards.

To investigate the effect of PLA-PCB recycling and subsequent extraction on the structure of PLA, FTIR spectra of primary PLA and recovered PLA-PCB were analyzed (Fig. 5). PLA shows characteristic stretching frequencies for C=O, –CH3 asymmetric, –CH3 symmetric, and C–O, at 1746, 2995, 2946, and 1080 cm^−1^, respectively. Bending frequencies for –CH3 asymmetric and –CH3 symmetric have been identified at 1452 and 1361 cm^−1^, respectively. The PLA recovered after single (Fig. 5b) and triple (Fig. 5c) recycling of PLA-PCB shows the same absorption peaks as the original PLA. Thus, the binder (PLA) has not undergone any chemical degradation during either the manufacture or recycling of PLA-PCB and can be reused for PCB manufacture.

Overall, after the recycling process of the lab-made PLA-PCB, 95% by weight of the binder (PLA), 100% by weight of the filler (fiberglass), 100% of the electronic components, and 100% of the copper conductors were recovered for reuse. According to average estimates, more than 50 million tons of e-waste are produced annually in the world, and WPCBs is 3–10 wt% of this mass^2^. Considering the average Cu content in WPCBs, it can be estimated that approximately 195,000 to 650,000 tons of copper are recycled or landfilled annually. In the case of 100% copper recycling with WPCBs, the economic effect would be from $1.6 billion and $5.38 billion annually.

The difference between laboratory-produced PLA circuit boards and circuit boards currently produced on an industrial scale (e.g., FR2 and FR4^31^) consists in the replacement of toxic and difficult-to-recycle binders based on epoxy and phenol–formaldehyde resins with an environmentally friendly one and easily recyclable PLA-based binder. The cost of the binder currently used in the industry to produce commercial PCBs varies from US$4.3 to US$4.7 per kilo^32^. The cost of PLA also ranges from $0.94 to $3.3 per kilo^33^. Therefore, the cost of the binders currently used in PCB manufacturing and the cost of PLA is in the same price range and the use of PLA as a binder for PCB fabrication will not increase the cost of the final product. In addition, a PLA-based PCB offers full recovery of raw materials and chemicals after the recycling process, which is currently unattainable with traditional PCBs^34^. An important advantage of the proposed PLA-PCB is that the PLA-based binder biodegrades without contaminating the environment with decomposition products when it ends up in a landfill^1^.

## Conclusions

Thus, we proposed a new process for PCBs manufacturing and recycling using PLA as a binder for efficient and eco-friendly recycling of WPCB. The novelty of the method consists in replacing the toxic and difficult to recycle thermosetting resins currently used for PCB production with PLA, which is biodegradable and easily recyclable. The study found that PLA-PCB can be easily recycled to its original components. Ultimately, after recycling the lab-made PLA PCB, more than 95% of the weight of the raw materials and 100% of the weight of the electronic components can be recovered for reuse. The PCB industry is currently based on overexploitation of non-renewable resources and is characterized by low recycling of WPCBs, which does not comply with the principles of a sustainable economy and ultimately increases the final price. From this point of view, gradually switching to renewable raw materials in the production of commercial PCBs and implementing processes for their easy recycling can positively impact the conservation of valuable, non-renewable resources and the possibility of their reuse. Implementing the manufacturing and recycling of PLA-PCB proposed in this article could promote the PCB manufacturing industry significantly closer to adopting a circular economy.

## Data Availability

All data generated or analyzed during this study are included in this published article.

## Abbreviations

E-waste: Electronic waste
PCB: Printed circuit board
WPCB: Printed circuit board waste
PLA: Polylactic acid
SEM: Scanning electron microscopy
FTIR: Four transform infrared spectroscopy
DSC: Differential scanning calorimetry
PLA-PCB: Laboratory-made composite material composed of woven fiberglass cloth with a PLA binder
FR2: Industrial composite material made of paper impregnated with a plasticized phenol–formaldehyde resin
FR4: Industrial composite material composed of woven fiberglass cloth with an epoxy resin binder
